# Factors associated with polypharmacy and the high risk of medication-related problems among older community-dwelling adults in European countries: a longitudinal study

**DOI:** 10.1186/s12877-022-03536-z

**Published:** 2022-11-07

**Authors:** Lizhen Ye, Junwen Yang-Huang, Carmen Betsy Franse, Tomislav Rukavina, Vanja Vasiljev, Francesco Mattace-Raso, Arpana Verma, Tamara Alhambra Borrás, Tasos Rentoumis, Hein Raat

**Affiliations:** 1 Department of Public Health, Erasmus MC, University Medical Center Rotterdam, P.O, . Box 2040, 3000 CA Rotterdam, The Netherlands; 2 Department of Social Medicine and Epidemiology, Faculty of Medicine, University of Rijeka, Rijeka, Croatia; 3 Department of Microbiology, Teaching Institute of Public Health of Primorsko-Goranska, County, Rijeka, Croatia; 4 Department of Internal Medicine, Erasmus MC, University Medical Center, Rotterdam, The Netherlands; 5 Epidemiology and Public Health Group, Division of Population Health, Health Services Research and Primary Care, School of Health Sciences, Faculty of Biology, Medicine and Health, Manchester Academic Health Sciences Centre, The University of Manchester, Manchester, UK; 6 Polibienestar Research Institute - Universitat de València ES, Valencia, Spain; 7 Alliance for Integrated Care, Athens, Greece

**Keywords:** Polypharmacy; Medication-related problems; Older people; Sex; Migration background, BMI, frailty, outpatient services, Health related quality of life

## Abstract

**Background:**

Polypharmacy can be defined as using five or more medications simultaneously. “Medication-related problems”, an extension of polypharmacy, includes inappropriate prescribing, poor adherence, overdosage, underdosage, inappropriate drug selection, inadequate monitoring, adverse drug effects, and drug interactions. Polypharmacy and the high risk of medication-related problems among older people are associated with adverse health consequences due to drug-drug interactions, drug-disease interactions, and adverse drug effects. This study aims to assess the factors associated with polypharmacy and the high risk of medication-related problems among community-dwelling older people in the Netherlands, Greece, Croatia, Spain, United Kingdom.

**Method:**

This longitudinal study used baseline and follow-up data from 1791 participants of the Urban Health Center European project. Polypharmacy and the risk of medication-related problems were evaluated at baseline and follow-up using the Medication Risk Questionnaire. We studied factors in the domains (a) sociodemographic characteristics, (b) lifestyle and nutrition, and (c) health and health care use. Hierarchical logistic regression analyses were used to examine the factors associated with polypharmacy and the high risk of medication-related problems.

**Results:**

Mean age was 79.6 years (SD ± 5.6 years); 60.8% were women; 45.2% had polypharmacy, and 41.8% had a high risk of medication-related problems. Women participants had lower odds of polypharmacy (OR = 0.55;95%CI:0.42–0.72) and a high risk of medication-related problems (OR = 0.50; 95%CI:0.39–0.65). Participants with a migration background (OR = 1.67;95%CI:1.08–2.59), overweight (OR = 1.37; 95%CI:1.04–1.79) and obesity (OR = 1.78;95%CI:1.26–2.51) compared to ‘normal weight’, with lower physical HRQoL (OR = 0.96, 95%CI:0.95–0.98), multi-morbidity (OR = 3.73, 95%CI:2.18–6.37), frailty (OR = 1.69, 95%CI:1.24–2.30), visited outpatient services (OR = 1.77, 95%CI: 1.09–2.88) had higher odds of polypharmacy. The associations with the high risk of medication-related problems were similar.

**Conclusions:**

Multiple factors in demography, lifestyle, nutrition, and health care use are associated with polypharmacy and the high risk of medication-related problems. Polypharmacy is a single element that may reflect the number of medications taken. The broader content of medication-related problems should be considered to assess the context of medication use among older people comprehensively. These provide starting points to improve interventions to reduce polypharmacy and high risk of medication-related problems. In the meantime, health professionals can apply these insights to identify subgroups of patients at a high risk of polypharmacy and medication-related problems.

**Trial registration:**

The intervention of the UHCE project was registered in the ISRCTN registry as ISRCTN52788952. The date of registration is 13/03/2017.

**Supplementary Information:**

The online version contains supplementary material available at 10.1186/s12877-022-03536-z.

## Background

People worldwide are living longer nowadays, but generally not in full health [1]. Living longer might result in people experiencing a period of life characterized by multi-morbidity [2]. Along with the multi-morbidity, the increasing number of medications used and the potentially inappropriate medication use among older adults have become a major health care concern [3].

Polypharmacy can be defined as using five or more medications simultaneously [4]. Medication-related problems, an extension of polypharmacy, can be defined as events or situations involving drug therapy that actually or potentially interferes with desired health outcomes [5]. It includes inappropriate prescribing, poor adherence, overdosage, underdosage, inappropriate drug selection, inadequate monitoring, adverse drug effects, and drug interactions [6, 7]. Hoel et al*.* [8] stated that, in general, using a higher number of medications is associated with the risk of more adverse drug events (ADEs), non-adherence, and costs. They suggested that patients taking five medications generally have a significant drug problem [8]. Polypharmacy and the high risk of medication-related problems among older persons are associated with increased health care costs, hospital admissions, and adverse health consequences, including falls, cognitive impairment and reduced quality of life [4, 9, 10]. Large-scale studies have estimated that 50.1 per 1000 person-years in ambulatory older adults and 1.89 per 100 person-months in institutionalized older community-dwelling adults have experienced adverse medication events [11, 12]. However, half of the observed adverse drug events could be prevented [11, 12]. Therefore, uncovering the associated factors related to polypharmacy and the high risk of medication-related problems becomes an essential step before taking action to prevent medication-related adverse events.

Studies have assessed which factors are associated with a higher risk of polypharmacy [13–18]. People with polypharmacy are generally older [13] and have a lower educational level than people without polypharmacy [14]. Former smokers have a higher risk of polypharmacy than persons who did not smoke [15]. Factors regarding health and health care use (e.g. falling, frailty, hospital admission, outpatient services use) have also been reported to be associated with a higher risk of polypharmacy [16, 17]. However, relatively few studies evaluated how a comprehensive set of relevant factors (i.e. sociodemographic, lifestyle and nutrition, as well as health and health care use) is associated with polypharmacy; also, relatively few studies assess the factors associated with the high risk of medication-related problems [18, 19]. Hence, our study assesses which factors in the domains of (a) sociodemographic characteristics, (b) lifestyle and nutrition, and (c) health and health care are associated with polypharmacy and the high risk of medication-related problems among community-dwelling older people in Europe. Moreover, this study aims to observe whether the associated factors regarding polypharmacy and the high risk of medication-related problems are similar and show a new insight to health care practitioners on preventing medication-related adverse events.

## Methods

### Study setting and population

This study was performed within the framework of the Urban Health Centres Europe (UHCE) project. The project was conducted in five countries (the Netherlands, Greece, Croatia, Spain, United Kingdom) between May 2015 and June 2017 [20]. The project aimed to prompt healthy ageing by approaches including a preventive multidimensional health assessment and integrated care pathways on appropriate medication prescription and adherence, prevention of fall risk, loneliness and frailty [21]. A total of 2325 participants who lived independently and could participate in the study for at least 6 months were recruited; 1215 were assigned to the integrated care pathway intervention; 1110 were assigned to the control group, which applied the “care as usual” [21]. All cities have followed ethical committee procedures, and approvals have been provided. Written informed consent was obtained from all participants. The study was registered as ISRCTN52788952. Further details on the interventions were described elsewhere [21, 22].

**Fig. 1:**
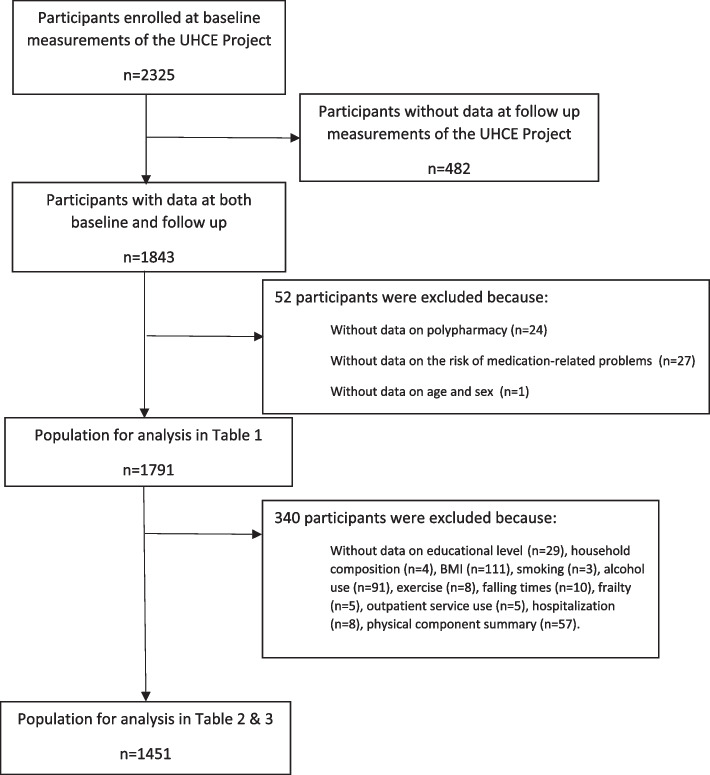
The flowchart of study population

In the current study, we adopted a longitudinal design and used baseline data and data after a 12-month follow-up of the UHCE project. Data was collected by self-reported questionnaires at both time points. In 2325 participants, participants who dropped out at follow-up (*n *= 482) were first excluded. Then, participants with missing data on polypharmacy (*n* = 24), the risk of medication-related problems (*n* = 27), and age and gender (*n *= 1) were further excluded. Thus, 1791 participants were included in this study. Due to the missing data on covariates, 340 participants were excluded from the main analysis (Fig. 1).

### Measurements

#### Outcomes

Polypharmacy and the risk of medication-related problems were evaluated at baseline and follow-up using the Medication Risk Questionnaire (MRQ) [7]. MRQ is a 10-item validated self-administered tool that can identify participants at a higher risk of medication-related problems, notably in the older population [7]. It covers polypharmacy, inappropriate prescribing, poor adherence, and multiple medical problems. Polypharmacy was measured by the question: Do you currently take five or more different medicines? [23] As shown in Supplementary Table S1, eight items of the MRQ were used to calculate the risk of experiencing medication-related problems. The 8-item MRQ was suggested by the original study of the questionnaire [7] and supported by a validation study [24]. The sum of item scores in the 8-item MRQ was used to indicate the risk level of experiencing medication-related problems. A risk score of 0 (lowest risk) to 8 (highest risk) indicates the level of risk [7]. Following the validating study on MRQ [24], participants were classified as a low-risk group if the total score is lower than 4 and a high-risk group if the score is equal to or larger than 4.

#### Associated factors

Based on the literature [13–17], variables assessed at baseline from three domains were considered as associated factors: sociodemographic factors, factors regarding lifestyle and nutrition, factors regarding health and health care use.

#### Sociodemographic factors

Sociodemographic factors included age (in years), sex (women/men), education level, country of residence (the Netherlands/ Greece/ Croatia/ Spain/ the UK), migration background (yes/no), and household composition (living with others/living alone). The level of education was reported as the highest level of education attained by a participant. It was classified into three categories according to the International Standard Classification of Education (ISCED): primary or less (ISCED 0–1), secondary or equivalent (ISCED 2–5), and tertiary or higher (ISCED 6–8) [25]. A participant was reported as having a migration background when the country of residence was not the country of birth.

#### Factors regarding lifestyle and nutrition

Factors regarding lifestyle and nutrition included smoking (yes/no), alcohol use (yes/no), physical activity, malnutrition (yes/no) and body mass index (BMI). One single question (i.e. Do you smoke at present?) was used to assess whether a person was a current smoker (yes/no) [20]. Alcohol use was assessed by three items of the Alcohol Use Disorders Identification Test (AUDIT-C), with a score from 0 (lowest risk) to 12 (highest risk), indicating the level of risk [26]. The variable was dichotomized (≥ 3 in women and ≥ 4 in men) to indicate whether a person was a hazardous drinker or had active alcohol use disorder (yes/no) [26]. The frequency of physical activity was assessed through answers to a question from the Frailty Instrument of the Survey of Health, Ageing and Retirement in Europe (SHARE-FI). The SHARE-FI was developed based on the existing questionnaire from SHARE [27]. Participants were asked to indicate the frequency of activities requiring a low or medium energy levels, such as gardening, cleaning the car or going for a walk. Answer categories included ‘once a week or less’ and ‘more than once a week’ [28]. Malnutrition was measured with the Short Nutrition Assessment Questionnaire 65 + (SNAQ-65 +) [29], which included the index of unintentional weight loss, mid-upper arm circumference (MUAC) and appetite and functional status. If a person lost 6 kg (13lbs) or more during the last 6 months, or 3 kg (6½ lbs) or more during the last month, or has a MUAC < 25 cm, he/she was classified as malnutrition. If a person had a poor appetite last week and difficulties climbing a staircase, he/she was classified as at risk of malnutrition [29]. BMI was calculated using self-reported height and weight (kg/m2). Participants were classified as underweight /normal weight (< 24.9 kg/m^2^), overweight (25–29.9 kg/m^2^) and obese (≥ 30 kg/m2), following WHO guidelines [28].

#### Factors regarding health and health care use

Health-related quality of life (HRQoL), multi-morbidity (yes/no), number of falls during the last year, frailty (yes/no), use of outpatient service (yes/no) and hospitalization (yes/no) during the last year were grouped in the factors regarding health and health care use. HRQoL was measured by the 12-Item Short-Form Health Survey (SF-12) [30]. The SF-12 includes 12 items encompassing eight health domains compiled in the Physical Component Summary (PCS) and Mental Component Summary (MCS), both ranging from 0 (lowest) to 100 (highest health status) [31]. Multi-morbidity was defined as having two or more common chronic conditions. A list of 15 common chronic conditions was used to identify older patients with multi-morbidity [20, 32]. Patients were asked to indicate whether they have conditions: heart attack (myocardial infarction or coronary thrombosis or any other heart problem including congestive heart failure), high blood pressure or hypertension, high blood cholesterol, a stroke or cerebral vascular disease, diabetes or high blood sugar, chronic lung disease, asthma, arthritis, osteoporosis, cancer or malignant tumor, stomach or duodenal ulcer or peptic ulcer, Parkinson’s disease, cataract, hip fracture or femoral fracture, and other conditions which are not yet mentioned. Participants reported the number of falls during the past 12 months. The falling number was dichotomized into “none or once” and “twice or more”. Frailty was assessed with the Tilburg Frailty + Indicator (TFI), a validated questionnaire which contains 15 self-reported questions [33]. The overall frailty score is the sum of the 15 items (score range 0–15) in the questionnaire. Participants with a total score ≥ 5 were classified as frail [34]. Outpatient services use (yes/no) was assessed by whether the participant visited a general practitioner or specialist during the last 12 months. Hospitalization (yes/no) was assessed by whether the participant had been admitted to the hospital during the last 12 months.

#### Statistical analyses

Descriptive statistics were used to describe the characteristics of the study population. Means and standard deviation (SD) were used to summarize the continuous variables and frequencies and percentages for categorical variables. Characteristics of the participants were compared according to polypharmacy and the risk of medication-related problems by T-test for continuous variables and chi-square tests for categorical variables.

Hierarchical logistic regression analyses were used to estimate longitudinal associations between the factors and polypharmacy or the high risk of medication-related problems. This approach is used to observe the effects of added variables and the change of effects on the risk of polypharmacy and the “risk of medication-related problems” at each step. Four separate analyses were done. In model 1, all sociodemographic factors at baseline were entered; in model 2, factors regarding lifestyle and nutrition at baseline were additionally added; in model 3, factors regarding health and health care use at baseline were additionally added; in model 4, polypharmacy (yes/no) or the variable “risk of medication-related problems” at baseline was further added to see the impact of the change in the outcomes. Whether the participants were divided into an intervention group or not (yes/no) was included as a covariate in all models. Odds ratios (OR) and 95% confidence intervals (95%CI) were calculated for each factor. *P*-values < 0.05 were considered statistically significant. The multi-collinearity test was performed to determine the correlation between the independent variables using a variance inflation factor (VIF). Collinearity exists when a VIF value is greater than 10 [35]. All analyses were conducted in the IBM SPSS Statistics for Windows, version 25 Armonk, NY, USA: IBM Corp.

#### Non-response analysis

The response group was defined as participants with full information (*n* = 1451). The non-response group was defined as participants with missing values (*n *= 874).

## Results

Table 1 presents the characteristics of the study population at baseline. The mean age was 79.6 years (SD ± 5.6 years), and 60.8% were women. Of all participants, 45.2% had polypharmacy, and 41.8% were classified as having a high risk of medication-related problems.

**Table 1:** Baseline characteristics of the study sample (*n* = 1791) by polypharmacy (no /yes) and the risk of medication-related problems (low risk /high risk) measured at follow-up

	**Total **^**b**^** (*n* = 1791)**	**Polypharmacy at follow-up** ^f^			**The risk of medication-related problems at follow-up** ^f^		
		**No *n* = 981 (54.8%)**	**Yes *n* = 810 (45.2%)**	***p*-value** ^a^	**Low risk *n* = 1043 (58.2%)**	**High risk *n* = 748 (41.8%)**	***p*-value** ^a^
**Sociodemographic factors**							
**Age** (Year)	79.6 ± 5.6	79.0 ± 5.7	80.2 ± 5.3	** < 0.001** ^d^	79.7 ± 5.9	79.4 ± 5.0	0.20 ^d^
**Sex** N(%)							
Female	1089 (60.8)	616 (62.8)	473 (58.4)	0.06 ^e^	667 (64.0)	422 (56.4)	**0.001** ^e^
Male	702 (39.2)	365 (37.2)	337 (41.6)		376 (36.0)	326 (43.6)	
**Educational level** N(%)							
Primary or less	446 (24.9)	261 (27.1)	185 (23.2)	0.06 ^e^	244 (23.8)	202 (27.4)	0.23 ^e^
Secondary	1142 (63.8)	601 (62.3)	541 (67.8)		677 (66.0)	465 (63.1)	
Tertiary or higher	174 (9.7)	102 (10.6)	72 (9.0)		104 (10.1)	70 (9.5)	
**Country** N(%)							
The Netherlands	272 (15.2)	135 (13.8)	137 (16.9)	** < 0.001** ^e^	157 (15.1)	115 (15.4)	**0.001** ^e^
Greece	259 (14.5)	171 (17.4)	88 (10.9)		161 (15.4)	98 (13.1)	
Croatia	427 (23.8)	256 (26.1)	171 (21.1)		278 (26.7)	149 (19.9)	
Spain	395 (22.1)	252 (25.7)	143 (17.7)		224 (21.5)	171 (22.9)	
The United Kingdom	438 (24.5)	167 (17.0)	271 (33.5)		223 (21.4)	215 (28.7)	
**Migration Background** N(%)							
No	1640 (91.6)	907 (92.8)	730 (90.1)	**0.045** ^e^	968 (92.8)	672 (89.8)	**0.03** ^e^
Yes	151 (8.4)	71 (7.2)	80 (9.9)		75 (7.2)	76 (10.2)	
**Household composition** N(%)							
Living with others	1106 (61.8)	635 (65.1)	471 (58.4)	**0.004** ^e^	654 (63.2)	452 (60.5)	0.25 ^e^
Living alone	676 (37.7)	340 (34.9)	336 (41.6)		381 (36.8)	295 (39.5)	
**Factors regarding life style and nutrition**							
**Smoking** N(%)							
No	1652 (92.2)	899 (92.0)	753 (93.2)	0.35 ^e^	955 (91.7)	697 (93.7)	0.12 ^e^
Yes	133 (7.4)	78 (8.0)	55 (6.8)		86 (8.3)	47 (6.3)	
**Alcohol use** N(%)							
No	1244 (69.5)	660 (71.7)	584 (75.5)	0.08 ^e^	707 (71.8)	537 (75.8)	0.06 ^e^
Yes	449 (25.1)	260 (28.3)	189 (24.5)		278 (28.2)	171 (24.2)	
**Exercise** N(%)							
More than once a week	1298 (72.5)	758 (77.8)	540 (67.2)	** < 0.001** ^e^	774 (74.8)	524 (70.5)	**0.046** ^e^
Once a week or less	480 (26.8)	216 (22.2)	264 (32.8)		261 (25.2)	219 (29.5)	
**Malnutrition** N(%)							
No	1516 (84.6)	833 (85.1)	683 (85.1)	0.99 ^e^	888 (85.3)	628 (84.8)	0.75 ^e^
Yes	266 (14.9)	146 (14.9)	120 (14.9)		153 (14.7)	113 (15.2)	
**BMI **^**c**^ N(%)							
Underweight and normal	607 (33.9)	360 (38.5)	247 (33.3)	**0.001** ^e^	386 (39.1)	221 (32.1)	** < 0.001** ^e^
Overweight	740 (41.3)	421 (45.1)	319 (43.0)		440(44.6)	300 (43.5)	
Obese	328 (18.3)	153 (16.4)	175 (23.6)		160 (16.2)	168 (24.4)	
**Factors regarding health and health care use**							
**Health-related quality of life (SF-12)** (score)							
Physical Component Summary	42.0 ± 11.9	44.9 ± 11.0	38.4 ± 12.0	** < 0.001** ^d^	43.6 ± 11.7	39.7 ± 11.9	** < 0.001** ^d^
Mental Component Summary	50.3 ± 10.7	50.8 ± 10.4	49.7 ± 11.0	**0.03** ^d^	50.4 ± 10.7	50.1 ± 10.7	0.52 ^d^
**Multi-morbidity** N(%)							
No	158 (8.8)	137 (14.0)	21 (2.6)	** < 0.001** ^e^	124 (11.9)	34 (4.5)	** < 0.001** ^e^
Yes	1629 (91.0)	840 (86.0)	789 (97.4)		915 (88.1)	714 (95.5)	
**Falling times (during the last year)** N(%)							
Zero or one time	1522 (85.0)	859 (88.6)	663 (82.4)	** < 0.001 **^e^	899 (87.3)	623 (83.6)	**0.03** ^e^
Two or more times	253 (14.1)	111 (11.4)	142 (17.6)		131 (12.7)	122 (16.4)	
**Frailty** N(%)							
No	804 (44.9)	516 (52.9)	288 (35.6)	** < 0.001** ^e^	525 (50.6)	279 (37.4)	** < 0.001** ^e^
Yes	979 (54.7)	459 (47.1)	520 (64.4)		513 (49.4)	466 (62.6)	
**Outpatient service use (during the last year)** N(%)							
No	153 (8.5)	113 (11.7)	40 (5.0)	** < 0.001** ^e^	120 (11.7)	33 (4.4)	** < 0.001** ^e^
Yes	1620 (90.5)	856 (88.3)	764 (95.0)		909 (88.3)	711 (95.6)	
**Hospitalization (during the last year)** N(%)							
No	1448 (80.8)	832 (85.6)	616 (76.9)	** < 0.001** ^e^	872 (84.6)	576 (77.6)	** < 0.001** ^e^
Yes	325 (18.1)	140 (14.4)	185 (23.1)		159 (15.4)	1661661661661661661662.4)	

^a^Presented as mean ± SD for continuous variables or N (%) for categorical variables; Significant *P*-values (< 0.05) are in bold

^b^Missing items: Educational level = 29 (1.6%); Household composition = 9 (0.5%); Smoking = 6 (0.3%); Alcohol use = 98 (5.5%); Exercise = 13 (0.7%); Malnutrition = 9 (0.5%); BMI = 116(6.1%); Multi-morbidity = 4 (0.2%);Frailty = 8 (0.4%); Falling times during the last year = 16 (0.9%); Outpatient service = 18 (1.0%); Hospitalization = 18 (1.0%)

^c^Abbreviations: BMI, body mass index

^d^p-values based on independent T test for continuous variables

^e^p-values based on chi-square test for categorical variables

^f^The sample size for polypharmacy and the risk of medication-related problems are both *n* = 1791

Polypharmacy was more frequently seen among older participants (*P* < 0.001) and participants living alone (*P* = 0.004). Women had a lower probability of high risk of medication-related problems than men (*P* = 0.001). Participants with a migration background had a higher probability of polypharmacy (*P* = 0.045) and high risk of medication-related problems (*P* = 0.03) than participants without a migration background. In general, the UK had the lowest probability of polypharmacy and high risk of medication-related problems, while Greece had the highest probability (*P* < 0.001). Participants with polypharmacy (*P* < 0.001) or with a high probability of high risk of medication-related problems (*P* = 0.046) were less likely to engage in physical activity more than once a week and more subject to being overweight (*P* < 0.01). The probability of polypharmacy or high risk of medication-related problems was higher in participants with lower physical HRQoL, experiencing multi-morbidity, and had fallen twice or more during the last year (all *P* < 0.05). The percentages of being frail, having visited outpatient services, and having been admitted to hospital during the past year were higher in participants with polypharmacy and at high risk of medication-related problems than their counterparts (all *P* < 0.001).

Table 2 presents the results from the hierarchical logistic regression about polypharmacy. For each model, multi-collinearity between factors was within acceptable limits (all VIF < 2). After adjusting for all potential factors (model 3), female sex (OR = 0.55, 95%CI: 0.42–0.72) was associated with a lower risk of polypharmacy. Participants from Greece (OR = 0.43, 95%CI: 0.26–0.71), Croatia (OR = 0.42, 95%CI: 0.28–0.63) and Spain (OR = 0.56, 95%CI: 0.36–0.8) had a lower risk of polypharmacy compared to participants from the Netherlands. Having a migration background (OR = 1.67, 95%CI: 1.08–2.59), being overweight (OR = 1.37, 95%CI: 1.04–1.79) or being obese (OR = 1.78, 95%CI: 1.26–2.51) were associated with a higher risk of polypharmacy. A higher level of physical HRQoL (OR = 0.96, 95%CI: 0.95–0.98) was associated with a lower risk of polypharmacy. Participants with multi-morbidity (OR = 3.73, 95%CI: 2.18–6.37), being frail (OR = 1.69, 95%CI: 1.24–2.30), or having visited outpatient services during the last year (OR = 1.77, 95%CI: 1.09–2.88) had a higher risk of polypharmacy than counterparts.

**Table 2:** Hierarchical logistic longitudinal regression models fitted on polypharmacy at follow-up (*n* = 1451^a^)

	**Polypharmacy at follow-up (Model 1) **^**d**^		**Polypharmacy at follow-up (Model 1) **^**e**^		**Polypharmacy at follow-up (Model 3) **^**f**^		**Polypharmacy at follow-up (Model 4) **^**g**^	
	**OR (95%CI) **^**b**^	***p*-value**^**c**^	**OR (95%CI) **^**b**^	***p*-value**^**c**^	**OR (95%CI) **^**b**^	***p*-value**^**c**^	**OR (95%CI) **^**b**^	***p*-value**^**c**^
**Intervention (yes)**	1.10 (0.89–1.37)	0.37	1.16 (0.93–1.45)	0.19	1.19 (0.94–1.50)	0.16	1.15 (0.87–1.53)	0.33
**Sociodemographic factors at baseline**								
**Age** (year)	1.02 (1.00–1.04)	0.09	1.02 (0.99–1.04)	0.14	0.99 (0.97–1.02)	0.58	0.99 (0.97–1.02)	0.68
**Sex** (female)	**0.78 (0.62–0.99)**	**0.045**	**0.73 (0.57–0.94)**	**0.01**	**0.55 (0.42–0.72)**	** < 0.001**	0.75 (0.54–1.04)	0.09
**Education level**		**0.04**		0.12		0.41		0.54
Tertiary or higher (ref.)	1.00		1.00		1.00		1.00	
Secondary	**1.63 (1.05–2.51)**	**0.03**	1.49 (0.96–2.33)	0.08	1.21 (0.80–1.85)	0.37	1.33 (0.80–2.21)	0.27
Primary	1.15 (0.78–1.7)	0.48	1.11 (0.75–1.66)	0.60	1.38 (0.86–2.21)	0.18	1.26 (0.71–2.22)	0.43
**Country**		** < 0.001**		** < 0.001**		** < 0.001**		** < 0.001**
The Netherlands (ref.)	1.00		1.00		1.00		1.00	
Greece	**0.59(0.38–0.92)**	**0.02**	**0.50 (0.32–0.78)**	**0.002**	**0.43 (0.26–0.71)**	**0.001**	**0.45 (0.24–0.82)**	**0.01**
Croatia	0.72 (0.51–1.03)	0.07	**0.58 (0.4–0.83)**	**0.003**	**0.42 (0.28–0.63)**	** < 0.001**	**0.47 (0.28–0.77)**	**0.003**
Spain	**0.58 (0.39–0.85)**	**0.006**	**0.56 (0.38–0.83)**	**0.004**	**0.55 (0.36–0.84)**	**0.005**	0.68 (0.41–1.14)	0.15
The United Kingdom	**1.62 (1.13–2.31)**	**0.008**	**1.7 (1.18–2.44)**	**0.004**	**1.52 (1.03–2.25)**	**0.03**	1.27 (0.80–2.04)	0.31
**Migration background** (yes)	1.48 (0.98–2.23)	0.06	**1.55 (1.02–2.35)**	**0.04**	**1.67 (1.08–2.59)**	**0.02**	1.56 (0.91–2.65)	0.10
**Living composition** (living alone)	1.26 (0.99–1.60)	0.06	1.23 (0.96–1.58)	0.10	1.14 (0.87–1.48)	0.35	1.04 (0.76–1.43)	0.81
**Factors regarding life style and nutrition at baseline**								
**Smoke** (yes)			1.09 (0.71–1.66)	0.70	1.01(0.65–1.57)	0.97	1.16 (0.68–1.97)	0.60
**Alcohol risk** (yes)			**0.76 (0.59–0.98)**	**0.03**	0.88 (0.67–1.16)	0.37	0.84 (0.60–1.17)	0.30
**Exercise** (once a week or less)			**1.75 (1.35–2.27)**	** < 0.001**	1.06 (0.79–1.43)	0.68	1.06 (0.74–1.51)	0.77
**BMI **^**b**^				** < 0.001**		**0.003**		**0.03**
Underweight and normal (ref.)			1.00		1.00		1.00	
Overweight			**1.47 (1.14–1.9)**	**0.003**	**1.37 (1.04–1.79)**	**0.02**	**1.40 (1.01–1.93)**	**0.045**
Obese			**1.99 (1.44–2.74)**	** < 0.001**	**1.78 (1.26–2.51)**	**0.001**	**1.70 (1.12–2.59)**	**0.01**
**Malnutrition** (yes)			0.97 (0.7–1.34)	0.83	0.76 (0.53–1.08)	0.12	0.71 (0.46–1.08)	0.11
**Factors regarding health and health care use at baseline**								
**Health-related quality of life (SF-12)** (score)								
Physical Component Summary					**0.96 (0.95–0.98)**	** < 0.001**	**0.98 (0.97- 0.996)**	**0.01**
Mental Component Summary					0.99 (0.98–1.00)	0.17	0.99 (0.98–1.01)	0.41
**Multi-morbidity** (yes)					**3.73 (2.18–6.37)**	** < 0.001**	**2.31 (1.24–4.31)**	**0.01**
**Falling times during the last year** (two or more times)					0.95 (0.68–1.32)	0.74	0.82 (0.55–1.24)	0.34
**Frailty** (yes)					**1.69 (1.24–2.30)**	**0.001**	1.44 (0.99–2.09)	0.05
**Outpatient service use during the last year** (yes)					**1.77 (1.09–2.88)**	**0.02**	1.24 (0.69–2.23)	0.47
**Hospitalization** (yes)					1.20 (0.88–1.63)	0.25	0.91 (0.63–1.33)	0.63
**Polypharmacy at baseline** (yes)							**15.91 (11.94–21.21)**	** < 0.001**
**Block**	75.47	** < 0.001**	45.44	** < 0.001**	147.40	** < 0.001**	**435.16**	** < 0.001**
**Model summary**	1918.15	0.95	1872.71	0.29	1725.31	0.69	1290.14	**0.03**
**Nagelkerke R**^**2**^	0.07		0.11		0.23		0.51	

^a^The number of missing cases is 340; the number of cases included in the analysis is 1451

^b^Abbreviations: BMI, body mass index; OR, odds ratio; CI, confidence interval

^c^Significant *P*-values (< 0.05) are in bold

^d^Model 1: Adjusted for intervention

^e^Model 2: Model 1 + Factors regarding life style and nutrition

^f^Model 3: Model 2 + Factors regarding health and health care use

^g^Model 4: Model 3 + Polypharmacy at baseline

Table 3 presents the results from the hierarchical logistic regression about medication-related problems. For each model, multi-collinearity factors were within acceptable limits (all VIF < 2). After adjusting for all potential factors (model 3), older age (OR = 0.96, 95%CI: 0.94–0.98) or female sex (OR = 0.50, 95%CI: 0.39–0.65) were associated with a lower risk of medication-related problems. Participants from Croatia (OR = 0.63, 95%CI: 0.41–0.98) were less likely to have a high risk of medication-related problems than those from the Netherlands. Having a migration background (OR = 1.81, 95%CI: 1.19–2.77), obesity (OR = 1.57, 95%CI: 1.13–2.19), having multi-morbidity (OR = 1.87, 95%CI: 1.19–2.93), being frail (OR = 1.75, 95%CI: 1.30–2.36), or having visited outpatient services during the last year (OR = 2.57, 95%CI: 1.54–4.29) were associated with the high risk of medication-related problems. A higher physical HRQoL (OR = 0.98, 95%CI: 0.96–0.99) was associated with a lower risk of medication-related problems.

**Table 3:** Hierarchical logistic longitudinal regression models fitted on the risk of medication-related problems at follow-up (*n* = 1451^a^)

	**The risk of medication-related problems at follow-up (Model 1) **^**d**^		**The risk of medication-related problems at follow-up (Model 2) **^**e**^			**The risk of medication-related problems at follow-up (Model 3) **^**f**^			**The risk of medication-related problems at follow-up (Model 4) **^**g**^	
	**OR (95%CI) **^**b**^	***p*-value**^**c**^	**OR (95%CI) **^**b**^	***p*-value**^**c**^		**OR (95%CI) **^**b**^		***p*-value**^**c**^	**OR (95%CI) **^**b**^	***p*-value**^**c**^
**Intervention (yes)**	1.01 (0.82–1.26)	0.90	1.04 (0.84–1.30)	0.72	1.04 (0.83–1.31)		0.71		0.97 (0.75–1.24)	0.79
**Sociodemographic factors at baseline**										
**Age** (year)	0.98 (0.96–1.00)	0.08	0.98 (0.96–1.00)	0.08		**0.96 (0.94–0.98)**		**0.001**	**0.96 (0.94–0.99)**	**0.004**
**Sex** (female)	**0.65 (0.52–0.83)**	** < 0.001**	**0.61 (0.48–0.78)**	** < 0.001**		**0.50 (0.39–0.65)**		** < 0.001**	**0.65 (0.49–0.87)**	**0.004**
**Education level**		**0.02**		0.05				0.12		0.18
Tertiary or higher (ref.)	1.00		1.00			1.00			1.00	
Secondary	**1.67 (1.09–2.56)**	**0.02**	**1.57 (1.02–2.41)**	**0.04**		1.18 (0.79–1.76)		0.43	1.24 (0.80–1.93)	0.34
Primary	1.12 (0.76–1.64)	0.57	1.10 (0.75–1.63)	0.63		1.54 (0.98–2.41)		0.06	1.57 (0.96–2.56)	0.07
**Country**		**0.01**		** < 0.001**				** < 0.001**		**0.003**
The Netherlands (ref.)	1.00		1.00			1.00			1.00	
Greece	0.73 (0.47–1.14)	0.16	0.66 (0.42–1.03)	0.07		0.68 (0.42–1.11)		0.12	0.72 (0.42–1.23)	0.23
Croatia	0.82 (0.57–1.17)	0.27	**0.68 (0.47–0.99)**	**0.04**		**0.59 (0.39–0.87)**		**0.008**	**0.63 (0.41–0.98)**	**0.04**
Spain	0.90 (0.62–1.33)	0.61	0.87 (0.59–1.28)	0.48		0.86 (0.57–1.29)		0.46	1.01 (0.64–1.58)	0.98
The United Kingdom	1.36 (0.95–1.94)	0.09	1.38 (0.96–1.98)	0.08		1.31 (0.90–1.90)		0.17	1.36 (0.90–2.06)	0.14
**Migration background** (yes)	**1.70 (1.13–2.55)**	**0.01**	**1.74 (1.15–2.61)**	**0.008**		**1.81 (1.19–2.77)**		**0.006**	**1.79 (1.12–2.84)**	**0.02**
**Living composition** (living alone)	1.27 (1.00–1.62)	0.06	1.24 (0.97–1.59)	0.08		1.14 (0.88–1.48)		0.31	1.12 (0.84–1.48)	0.45
**Factors regarding life style and nutrition at baseline**										
**Smoke** (yes)			0.91 (0.60–1.40)	0.68		0.84 (0.54–1.3)		0.43	1.04 (0.65–1.67)	0.87
**Alcohol risk** (yes)			**0.70 (0.54–0.90)**	**0.006**		0.77 (0.59–1.01)		0.06	0.80 (0.60–1.07)	0.14
**Exercise** (once a week or less)			1.27 (0.98–1.64)	0.07		0.89 (0.66–1.18)		0.41	0.93 (0.67–1.27)	0.63
**BMI **^**b**^				**0.002**				**0.03**		0.08
Underweight and normal (ref.)			1.00			1.00			1.00	
Overweight			**1.34 (1.04–1.72)**	**0.02**		1.23 (0.95–1.60)		0.12	1.17 (0.88–1.56)	0.29
Obese			1.77 (1.29–2.44)	** < 0.001**		**1.57 (1.13–2.19)**		**0.007**	**1.53 (1.06–2.20)**	**0.02**
**Malnutrition** (yes)			1.12 (0.81–1.54)	0.50		0.92 (0.66–1.30)		0.64	0.86 (0.59–1.50)	0.43
**Factors regarding health and health care use at baseline**										
**Health-related quality of life (SF-12)** (score)										
Physical Component Summary						**0.98 (0.96–0.99)**		** < 0.001**	0.99 (0.98–1.00)	0.14
Mental Component Summary						1.00 (0.99–1.02)		0.74	1.00 (0.99–1.02)	0.68
**Multi-morbidity** (yes)						**1.87 (1.19–2.93)**		**0.006**	1.50 (0.92–2.43)	0.10
**Falling times during the last year** (two or more times)						0.92 (0.67–1.28)		0.64	0.90 (0.62–1.29)	0.55
**Frailty** (yes)						**1.75 (1.30–2.36)**		** < 0.001**	**1.61 (1.16–2.23)**	**0.005**
**Outpatient service use during the last year** (yes)						**2.57 (1.54–4.29)**		** < 0.001**	**1.98 (1.13–3.48)**	**0.02**
**Hospitalization** (yes)						1.14 (0.84–1.54)		0.40	1.03 (0.74–1.43)	0.86
**The risk of medication-related problems at baseline** (high risk)									**6.83 (5.29–8.80)**	** < 0.001**
**Block**	40.19	** < 0.001**	26.74	** < 0.001**		94.37		** < 0.001**	241.77	** < 0.001**
**Model summary**	1926.28	0.55	1899.54	0.99		1805.17		0.24	1563.40	0.85
**Nagelkerke R**^**2**^	0.037		0.061			0.14			0.33	

^a^The number of missing cases is 340; the number of cases included in the analysis is 1451

^b^Abbreviations: BMI, body mass index; OR, odds ratio; CI, confidence interval

^c^Significant *P*-values (< 0.05) are in bold

^d^Model 1: Adjusted for intervention

^e^Model 2: Model 1 + Factors regarding life style and nutrition

^f^Model 3: Model 2 + Factors regarding health and health care use

^g^Model 4: Model 3 + The risk of medication-related problems at baseline

Supplementary Table S2 presents the association between polypharmacy and the high risk of medication-related problems in the study population. Compared with participants without polypharmacy at follow-up, participants with polypharmacy were more likely to have a high risk of medication-related problems (*P* < 0.001).

### Non-response analysis

Compared to the population-for-analysis (*n* = 1451), participants excluded from the study due to the missing data (*n* = 874) were more likely from the UK and less likely from Spain (*P* < 0.001), more likely to engage in physical activity once a week or less (*P* < 0.05), less likely having visited outpatient services during the past year (*P* < 0.001), more likely having been admitted to hospital during the past year (*P* < 0.05), more likely being malnutrition (*P* < 0.05).

## Discussion

The present study assessed the factors longitudinally associated with polypharmacy and the high risk of medication-related problems in a large sample of older community-dwelling people in Europe. Multiple factors (i.e. sex, migration background, HRQoL, multi-morbidity, BMI, frailty, and outpatient service use during the last year) were significantly associated with polypharmacy and the high risk of medication-related problems.

In the present study, no association was found between age and polypharmacy. This result is not in line with previous studies' findings, which reported that older people are more likely to have polypharmacy [13, 15]. A possible explanation could be the relatively high age of the participants in our study compared with previous studies. A nationwide report from Italy found that medication use increases steeply until age 85–90 years and then declines substantially [36]. The reason might be that medication prescribing and utilization are generally treated cautiously among very old people [37]. It may explain the absence of the association between age and polypharmacy in our study population with a mean age of 80. It may also explain that in our study, there was a negative association between age and a high risk of medication-related problems, which is consistence with the study from Katharina Tabea Jungo et al*.* [38]. Further studies are needed to explore the association between age and polypharmacy and the high risk of medication-related problems among older people.

We confirmed that women were less likely to have polypharmacy and were at a lower risk of medication-related problems than men [39]. The possible explanation could be that women are more likely to care about their health status; they consult doctors more regularly and earlier than men [39]. Therefore, women might accumulate more experience and knowledge on medication use than men of the same age. However, our result was inconsistent with findings from other studies [40, 41], which showed that the female sex was associated with an increased risk of medication-related problems. Therefore, the association between sex and the risk of medication-related problems is inconclusive, and more studies are needed.

Our results also showed that participants with a migration background were more likely to be at risk of polypharmacy and at high risk of medication-related problems. The explanation could be that participants with a migration background have personal beliefs about medication that rely on their background (e.g. cultural, core values, religious and health-related experience), which may differ from the healthcare providers of the country they reside in [42]. Additionally, the communication between participants with a migration background and healthcare providers could be challenging due to the possible language differences [43]. Furthermore, participants with a migration background might know less about the local health system (e.g. health insurance coverage, treatment process) and might experience barriers when receiving the appropriate health service, including medication prescribing [44]. Thus, services adapted to the population's specific needs with migration background, such as translated and simplified information on prescription use, may reduce polypharmacy and the high risk of medication-related problems [45].

We found that a higher physical HRQoL was associated with a lower risk of polypharmacy, as well as a lower risk of medication-related problems. Meanwhile, mental HRQoL was not significantly associated with polypharmacy or the high risk of medication-related problems. These findings are consistent with previous studies in the USA [46–48].

Our results showed that older people with multi-morbidity were more likely to have polypharmacy and a high risk of medication-related problems. Multiple medical specialists may treat people with multiple chronic conditions, and specialists may not know what medicines their patients are taking, which may lead to inappropriate prescribing [19, 49]. In addition, the high risk of medication-related problems and poor adherence by the patient may be due to a lack of adequate communication between prescribers and pharmacies [3]. Thus, better communication on each condition between patients and their specialists, between different specialists is essential for people with multiple chronic conditions.

Our study confirmed that frail older people are more likely to be at high risk of polypharmacy and the high risk of medication-related problems [38]. The explanation could be that frail people are more likely to have multi-morbidity [2, 28, 49].

We found that participants who used outpatient services during the last 12 months were more likely to have polypharmacy and have a high risk of medication-related problems. Hu et al. showed that more frequent access to outpatient services is associated with more prescriptions and a higher risk of medication-related problems [49].

Younger age, male sex, migration background, frailty, and outpatient services utilization during the last 12 months were negatively associated with the change in the risk of medication-related problems but not significantly associated with the change in polypharmacy risk. However, lower physical HRQoL and higher risk of multi-morbidity were significantly associated with the change in the risk of medication-related problems but not polypharmacy risk. The differences in the associations of the above factors on change of polypharmacy and the risk of medication-related problems are due to the information difference in which polypharmacy and medication-related problems are covered. Polypharmacy measures the number of medicines the participants take, which captures part of the medication-related problems a participant may encounter. On the other hand, MRQ offers a wider measure of the risk of medication-related problems, for example, information on following the prescription correctly. Further studies are needed to explore the association between the above factors and medication utilization.

### Methodological considerations

Our results need to be interpreted with caution. The population in this study is 75 years and older, so we cannot generalize our findings to the entire community-dwelling older population. The risk of medication-related problems were assessed by the self-reported questionnaire MRQ, which reflects different medication-related problems (e.g. poor adherence, inadequate monitoring). However, individual prescriptions or inappropriate drug selection were not assessed in the questionnaire. Therefore, caution is needed when interpreting the results.

## Conclusions

Multiple factors in demography, lifestyle, nutrition, and health care use are associated with polypharmacy and the high risk of medication-related problems. Polypharmacy is a single and important element that may reflect the number of medications taken. To comprehensively assess the context of medication use among older people, the broader content of medication-related problems should be considered as well. These provide starting points to improve interventions to reduce polypharmacy and the risk of medication-related problems. In the meantime, health professionals can apply these insights to identify subgroups of patients at a high risk of polypharmacy and having a high risk of medication-related problems.

## Supplementary Information

**Additional file 1: Supplementary Table S1.** 8 items on the risk of medication-risk questionnaire [1]. **Supplementary Table S2. **The associationbetween polypharmacy and the risk of medication-related problems in the studypopulation. (*n*=1791).

## Data Availability

The datasets analyzed during the current study are not publicly available due to privacy/ethical restrictions but are available from the corresponding author on reasonable request.

## Abbreviations

UHCE: Urban Health Centres Europe
MRQ: Medication Risk Questionnaire
ISCED: The International Standard Classification of Education
BMI: Body mass index
AUDIT-C: The Alcohol Use Disorders Identification Test
SNAQ-65 +: The Short Nutrition Assessment Questionnaire 65 +
MUAC: Mid-upper arm circumference
WHO: World Health Organization
HRQoL: Health-related quality of life
SF-12: The 12-Item Short-Form Health Survey
PCS: Physical Component Summary
MCS: Mental Component Summary
TFI: The Tilburg Frailty Indicator
SD: Standard deviation
OR: Odds ratios
95%CI: 95% Confidence intervals
VIF: Variance Inflation Factor
