# Supplementary material for: Factors associated with polypharmacy and the high risk of medication-related problems among older community-dwelling adults in European countries: a longitudinal study
Source: BMC Geriatr. 2022 Nov 7;22:841. doi: 10.1186/s12877-022-03536-z (PMC9641844; doi:10.1186/s12877-022-03536-z)
Supplement: Supplementary file 1 — Additional file 1: Supplementary Table S1. 8 items on the risk of medication-risk questionnaire [1]. Supplementary Table S2. The associationbetween polypharmacy and the risk of medication-related problems in the studypopulation. (n=1791). [file 12877_2022_3536_MOESM1_ESM.docx]

**Supplementary Table S1. 8 items on the risk of medication-Risk Questionnaire [1].**

| NO. | Questions | Answer Options |
| --- | --- | --- |
| 1 | Do you currently take 5 or more medications? | Yes/No |
| 2 | Do you take 12 or more medication doses per day? | Yes/No |
| 3 | Have your medicines or the instructions on how to take them been changed 4 times or more in the last 12 months? | Yes/No |
| 4 | Is it difficult for you to take your medicines as prescribed? | Yes/No |
| 5 | Does more than one doctor prescribe medicines for you on a regular basis? | Yes/No |
| 6 | Do you collect your own medicines from the pharmacy? | Yes/No |
| 7 | Are your prescriptions always dispensed at the same pharmacy? | Yes/No |
| 8 | Are you currently taking medicines for 3 or more medical problems? | Yes/No/I am unsure |

References

1. Barenholtz Levy, H., *Self-administered medication-risk questionnaire in an elderly population.* Ann Pharmacother, 2003. **37**(7-8): p. 982-7.

**Supplementary Table S2. The association between polypharmacy and the risk of medication-related problems in the study population. (n=1791).**

|  |  |  | The risk of medication-related problems | | |
| --- | --- | --- | --- | --- | --- |
|  |  | Total | Low | High | p-value |
| Polypharmacy | No | 981 (54.8%) | 868 (83.2%) | 113 (15.1%) | <0.001 |
|  | Yes | 810 (45.2&) | 175 (16.8%) | 635 (84.9%) |  |
|  | Total | 1791 | 1043 (58.2%) | 748 (41.8%) |  |

Notes: p-value is based on chi-square test.
